# Biliary Complications Postlaparoscopic Cholecystectomy: Mechanism, Preventive Measures, and Approach to Management: A Review

**DOI:** 10.1155/2011/967017

**Published:** 2011-06-12

**Authors:** Norman Oneil Machado

**Affiliations:** Department of Surgery, Sultan Qaboos University Hospital, P.O. Box 38, Muscat 123, Oman

## Abstract

Laparoscopic cholecystectomy has emerged as a gold standard therapeutic option for the management of symptomatic cholelithiasis. However, adaptation of LC is associated with increased risk of complications, particularly bile duct injury ranging from 0.3 to 0.6%. Occurrence of BDI results in difficult reconstruction, prolonged hospitalization, and high risk of long-term complications. Therefore, more emphasis is placed on preventing these complications. In addition to adequate training, several techniques have been proposed to prevent bile duct injury including use of 30° scope, adequate delineation of structures in Calot's triangle (critical view), avoidance of diathermy close to common hepatic duct, and intraoperative cholangiogram, and to maintain a low threshold to conversion to open approach when uncertain. Management of Bile duct injury depends on the nature of injury, time of detection, and the expertise available, and would range from simple subhepatic drainage to Roux-en-Y hepaticojejunostomy particularly performed at specialised centers. This article based on the literature review aims to review the biliary complications following laparoscopic cholecystectomy with reference to its mechanism , preventive measures to be taken, and the management approach.

## 1. Introduction

Laparoscopic cholecystectomy (LC) has replaced open surgery in the treatment of symptomatic cholecystolithiasis [1–8]. While LC offers the patient several advantages of minimal invasive surgery, the spectrum of complications in gallstone surgery has changed compared to open procedure. Laparoscopy-related complications such as bile duct injury (BDI) tend to be complex being more proximal and often associated with concomitant vascular injury [9]. This along with injuries during access into peritoneal cavity such as bowel and major retroperitoneal vascular injury has raised the morbidity to 2.9% [1–4]. The spectrum of mishap has also changed due to the involvement of new instruments such as stapling device and energized instruments. Related complications like migrating clips or spillage of gallstone into peritoneal cavity were completely unknown in open surgery. Surgical procedure used in the management of stricture include, Roux- en-Y hepaticojejunostomy, hepatectomy, and liver transplantation [3–6]. Recurrence of biliary stricture after a surgical repair can present many years later [5]. Therefore, these patients require long-term, may be life-long follow-up with hospital visits and investigations to detect recurrent stricture [5–8].

Patients who sustain BDI during cholecystectomy have impaired quality of life and continue to have a higher risk of dying as compared with those who have an uncomplicated cholecystectomy [7, 8]. There is a significant increase in healthcare expenses associated with the complication, and this is a common reason for medical malpractice litigation [7, 8]. Large compensation is often awarded to the patients in these instances. In this paper based on the literature review, biliary complications are discussed with reference to its mechanism, preventive measures, and management approach.

## 2. Risk Factors for BDI

Severe local risk factors that predispose to BDI have been reported such as acute cholecystitis, acute biliary pancreatitis, bleeding in Calot's triangle, severely scarred or shrunken gall bladder, large impacted gallstone in Hartmann's pouch, short cystic duct, and Mirizzi's syndrome. In addition, abnormal biliary anatomy is a common reason for BDI after LC [1–8]. Male sex and prolonged surgery for more than 120 minutes are reported to be independent risk factors [1]. However although local risk factors are reported to be present in 15–35% of BDI, more than half of all such injuries occurred during the so called “easy” LC performed by an inexperienced surgeon [1].

## 3. Impact of Biliary Complications

Acute BDI results in short-term complications such as biloma, bile peritonitis, sepsis, multiple organ dysfunction syndrome, external biliary fistula, cholangitis, liver abscess, and others [2–4, 6, 8]. These complications if not properly managed may be associated with mortality as high as 5% [10]. Laparoscopic cholecystectomy is also associated with a higher risk of vascular injury to the hepatic artery and portal vein which further increases the mortality [9].

Acute BDI and the ensuing biliary fistula may evolve into a biliary stricture. If the biliary stricture is not appropriately managed, the complications of intrahepatic lithiasis, secondary biliary cirrhosis, portal hypertension, and end stage liver disease may follow [3, 5, 8].

## 4. Investigative Approach

**Figure 1:**
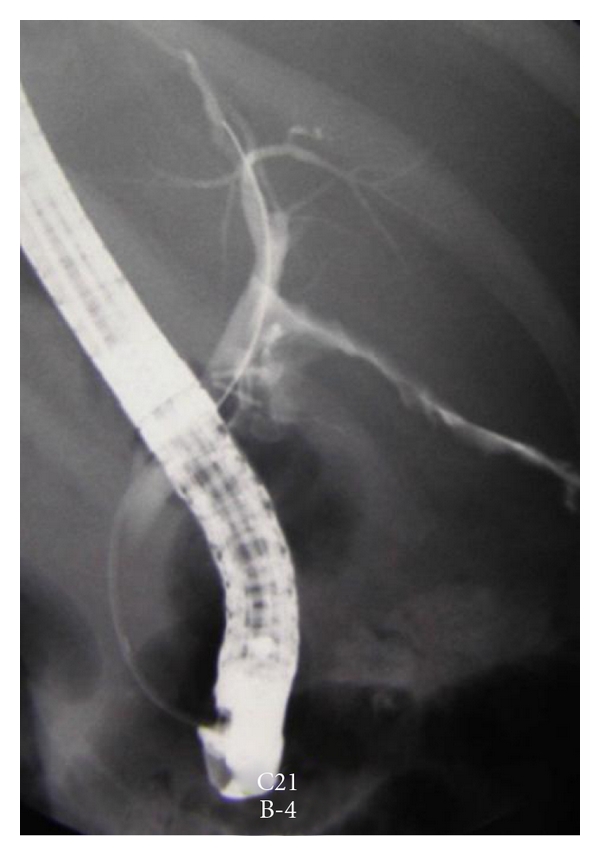
ERCP showing small CBD leak managed effectively by sphincterotomy.

**Figure 2:**
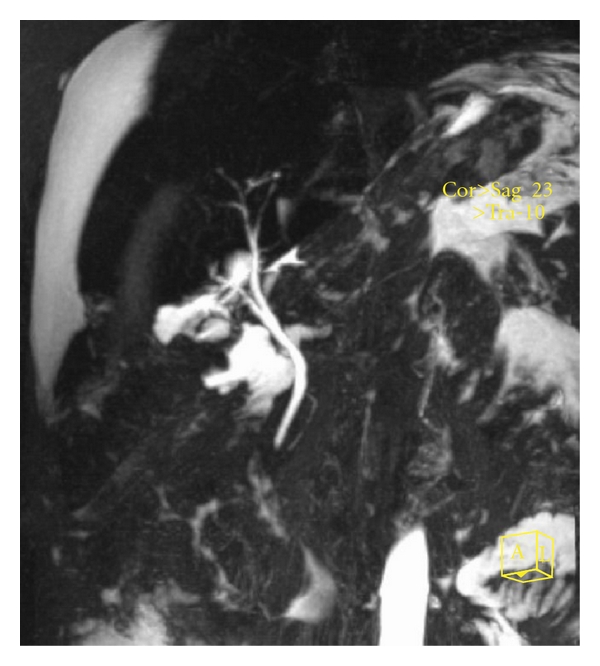
MRCP revealing subhepatic and significant intra-abdominal bile collection from cystic duct leak. The CBD is not dilated. The patient was managed effectively with ERCP sphincterotomy.

**Figure 3:**
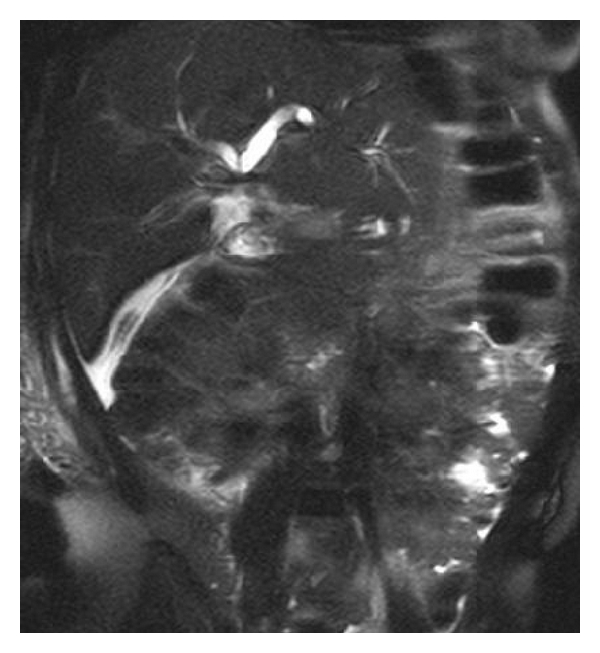
MRCP revealing a complete transection of CBD just below the hilum. Subhepatic and intraperitoneal collection can also be noted. Patient underwent a successful hepaticojejunostomy and continues to do well 4 years after surgery.

Abnormal liver function test suggestive of BDI should be investigated further. A wide array of imaging techniques is used to identify the nature and extent of injury and the associated complications [11]. Abdominal ultrasonography as the initial investigation of choice may demonstrate fluid collection within the right subhepatic space in addition to revealing a proximal dilated biliary system in patients with complete division of the CBD [11]. Further investigation is then warranted in the evaluation of the cause of this collection or suspected obstruction. In many cases, abdominal CT is unhelpful merely confirming the ultrasound appearance [11]. Endoscopic retrograde cholangiography (ERCP) (Figure 1) and magnetic resonance cholangiography (MRC) examination are likely to demonstrate the presence of biliary leak (Figure 2) and often provide the level of duct laceration or transaction [11] (Figure 3). ERCP in addition provides a therapeutic option in this scenario when sphincterotomy and endobiliary stenting may be considered [12]; other therapeutic intervention commonly used includes percutaneous transhepatic cholangiography, transhepatic biliary drainage, and percutaneous drainage of intra-abdominal collection [12]. Due to its excretion into the biliary tree h-imino-diacetic acid (HIDA), scintigraphy may be of value in investigation of patients with suspected biliary leak. It may also demonstrate continuity between the biliary tree and the subhepatic collection [11].

## 5. How to Avoid a Bile Duct Injury

Adequate and proper training in a laparoscopic surgery, delineation of biliary anatomy in Calot's triangle (critical view) by careful surgical dissection, and if need be by intra-operative cholangiography (IOC), judicious use of electrocautery, avoiding blind application of clips, and cautery in case of bleeding in the Calot's triangle are some of the measures to avoid a BDI [1, 2, 4, 6, 8, 10]. The primary cause of error according to one report was visual perceptual illusion in 97% of the cases [13]. Fault in technical skill was present in only 3% of injuries. Knowledge and judgment error contributed but were not the primary cause [13].

## 6. Correct Exposure and Identification of Structures in Calot's Triangle

The main cause of inadvertent transection of CBD in LC is mistaking CBD for cystic duct [1, 3, 4, 6, 13]. To avoid misidentification of CBD as the cystic duct, it is essential to visualize meticulously, in order to obtain the first impression of the extrahepatic bile duct, before dissection is started preferably using a 30° laparoscope. Some landmarks including cystic lymph node, gall bladder neck, and Rouviere's sulcus have been advocated for identifying the cystic duct and safe dissection [14]. Hartman's pouch is often used as a landmark as it is easily visualized and connects GB to cystic duct. Care, however, is taken in cases where it is distorted or abolished as in patients with atrophic cholecystitis, impacted cystic duct stone, adhesions between cystic duct, and the neck of gall bladder and in incorrect dissection [15]. In difficult cases, Rouviere's sulcus is a useful landmark which is visualized when the neck of GB is retracted upwards and towards the left exposing posterior aspect of hepatocystic triangle. Rouviere's sulcus is seen running to the right of the liver hilum anterior to the caudate lobe and indicates the plane of CBD accurately; a triangle bounded by the neck of GB, the liver surface, and the plane of sulcus is found and dissection can be started safely by division of the peritoneum immediately ventral to the sulcus [14, 16]. In addition to the anatomic landmark, traction on the gall bladder should be in a proper direction in identifying the cystic duct because of the 2-dimensional perspectives during LC. Mistaken identification of the CBD as cystic duct can be attributed to the direction of traction on the gall bladder in superior direction rather than laterally bringing the cystic duct and CBD into alignment [17]. When clipping or dividing the cystic duct, it is essential to retract the GB in lateral direction. In case of difficult cholecystectomy resulting from extensive adhesions, acute cholecystitis, long standing chronic cholecystitis, small contracted gall bladder, and fibrosed and obliterated Calot's triangle, early conversion from laparoscopic to open operation is advised [17]. An intraoperative cholangiogram (IOC) will delineate the anatomy in difficult cases though there are some who advise them in all cases of laparoscopic cholecystectomy [1]. When a reasonable period of time has been spent on trial dissection without significant progress, conversion to open cholecystectomy is the safest option [1, 3, 4, 8].

## 7. To Avoid Thermal Injury

Misuse of cautery in dissecting the Calot's triangle may cause serious BDI with loss of ductal tissue due to thermal necrosis [17]. Some of the measures used to avoid thermal injury to major bile duct include to initially hook through limited amount of tissue and lift the tissue off the underlying structures under precise vision and proceed with dissection. Once the serosa of the gall bladder is opened, blunt dissection using peanut may be employed instead of cautery in Calot's triangle [4]. In addition to being an effective alternative to cautery, it offers better field view particularly in cases of minor oozing [4, 17]. It is of outmost importance not to use cautery to cut the cystic duct particularly when titanium clips are placed on the cystic duct as titanium clips are good electrical conductor and may lead to thermal necrosis of the cystic duct stump or adjacent bile duct. It is pertinent that always short bursts of minimal amount of energy required to dissect or secure homeostasis should be applied [17].

## 8. To Avoid Blind Haemostasis

Dissection in the presence of acute inflammation and scarred tissue may result in significant bleeding obscuring the visualization of the biliary structure [4, 17]. A panic response in such situation will invariably lead to clipping or cauterization in areas inadequately exposed leading to increased risk of BDI or worse still a combination of vascular and bile duct injury [4, 17]. Bleeding in such situation is adequately dealt by maintaining a calm composure in addition to compressing the bleeding point and adjoining tissue with atraumatic forceps for several minutes [4, 17]. The haemorrhage is usually controlled in several minutes avoiding major bile duct injury by careless application of clips or cautery. Once good exposure is obtained, clips can be placed accurately. However, in the presence of uncontrolled bleeding, it is prudent to convert to open surgery.

## 9. Awareness of Anatomic Variation

Several anatomic variations of the biliary tract and hepatic vessel and its branches increase the risk of injury during LC particularly in the presence of acute inflammation. Measures to prevent injury include dissection of the cystic artery and duct as close as possible to the gall bladder and avoiding dissection of cystic duct to its termination into CBD as it is extremely dangerous in patients with low insertion of cystic duct. Cystic artery identification is achieved when a branch vessel of it can be clearly identified entering into GB. The hepatic artery is particularly at risk of being damaged in the presence of Moynihan hump, and the right hepatic duct is at risk when cystic duct occasionally opens directly into it. Opening the hepatobiliary triangle completely is required before any significant structure is divided which means that that 3-dimensional exposure and identification of the cystic infundibulum and cystic duct and artery is required. The 2 identified structures entering the gall bladder can only be the cystic duct and artery [4].

## 10. Conversion to Open Approach When Necessary

The conversion rates during LC vary from 3.6 to 13.9% [1, 4, 18]. The common indication for conversion includes technical difficulties, uncontrolled bleeding, difficulty in dissecting the Calot's triangle, CBD stones, and bile duct injuries [4, 17, 18]. In the event of uncertainty of anatomical landmarks and failure to progress after a reasonable period of dissection, one should not hesitate to convert for it reflects the sound judgment on the part of surgeon rather than failure to accomplish an otherwise difficult and hazardous task which may be detrimental to patients surgical outcome.

## 11. Role Intraoperative Cholangiogram and Laparoscopic Ultrasound

The role of routine intraoperative cholangiography (IOC) to avoid BDI remains controversial [1, 19, 20]. Although some series report excellent results for LC with little need for routine IOC, others report its use to decrease the incidence of BDI or its early detection intraoperatively [1, 19]. In one report, 81% of BDI were detected at the time of initial injury when IOC was carried out compared with only 45% when it was not employed [20]. However the disadvantage of IOC includes the need for surgical experience, the inevitable prolongation of the operative time and the need for interpretation by an experienced radiologist [4]. The use of laparoscopic ultrasound is another attractive alternative as it can be performed without any tissue dissection or cannulation in the biliary tract [21]. It is reported to be particularly useful in difficult cases because of inflammation or adhesions as it guides to identify the position of the cystic duct and the main extrahepatic bile ducts. It is considered to be reliable, fast, repeatable, and cost-effective method for identification of bile duct anatomy in difficult cases [21].

## 12. Surgeons Characteristics of Risk Taking Tendency and BDI

Analysis of the relationship of surgeons characteristics and risk taking preference to incidence of CBDI has revealed that surgeons with extreme risk taking preference (like those who are not bothered in taking risks if the gain involved are believed by them to be high compared to those who avoid situation with uncertain outcome) demonstrated a higher injury rate among those with high risk taking preference score [13, 22]. Inadvertent injuries of bile duct are reported to occur due to casual approach, overconfidence, and ignorance of difficult situations [4]. The surgical community is working towards creating a safety culture where even a rare event like BDI are accounted for by better training and standard use of safety measures. Some of these include incorporating modules that simulate potential intraoperative errors in judgment (like misidentification of CBD) and stress safe decision making and emphasize use of proved safety measures that are likely to be helpful. Surgical simulation in this regard is likely to become an integral part of training [22].

## 13. Management of Strategy When Faced with a Bile Duct Injury

Bile leak during cholecystectomy should force surgeon to stop and carefully examine the source of bile leak [3, 4, 6, 8, 17]. Although bile may leak from an opening in the GB or the cystic duct, before that is presumed to be the case, BDI should be ruled out. Bile from GB is greenish yellow, thick, and viscid, whereas common bile duct (CBD) bile usually is bright yellow, thin, and watery. An IOC at this stage may delineate the anatomy and prevent any further injury to the bile duct. A BDI should also be suspected if a third tubular structure (after cystic duct and artery have been clipped and divided) is encountered in the Calot's triangle. The “cystic duct” which was clipped and divided earlier may actually have been the CBD and the third structure now being encountered may be the common hepatic duct. If the BDI is recognized intraoperatively, the management depends on the nature of the duct injured, type of injury, and the expertise and experience of the surgeon [1, 4, 6, 8].

## 14. Approach to Specific Injuries to Bile Duct

### 14.1. Clipped Common Bile Duct

Occasionally, a clip may be placed incorrectly without the division of the bile duct. In such an event removal of clip may suffice [6]. If there is no perforation of the duct, nothing more needs to be done. If biliary obstruction is detected postoperatively in such a patient as suggested by elevated alkaline phosphatase levels, intrahepatic bile duct dilatation on imaging or delayed excretion of isotope on hepatobiliary scintigraphy, an endoscopic stent is placed [6].

### 14.2. Minor Duct

A surgeon often encounters minor ducts particularly while dissecting the gall bladder of its bed. These include cholecystohepatic duct, subvesical duct, and small (less than 3 mm) subsegmental duct in GB bed [6]. If a minor duct is injured, it may be clipped [6]. This will result in asymptomatic atrophy of a segment of liver [22]. If this is not feasible, draining the subhepatic fossa as described subsequently may be useful. However when bile leakage from the open end of the duct is noted intraoperatively, it is prudent to assess the nature of injury before it is assumed to be arising from an aberrant bile duct and leaking duct in the bed of GB or a leaking cystic duct stump, for ligation and suture in such situation may increase the severity of the injury and necessitate reoperation. It is imperative to define the anatomy of the biliary duct by cholangiography, and avoid any additional dissection that may further injure or devascularize the bile duct. Different methods may be used to evaluate the biliary duct for different types of injuries and different detection time of injuries [12]. For patients with intraoperative bile leakage, cholangiography via cystic duct or the open end of the duct can be done to determine which duct is injured and to assess the nature of injury. In patients suspected of duct injury in postoperative period, ERCP will show the site of transaction or leakage [12]; however, ERCP cannot show the proximal end of the bile duct when the duct is clipped divided or excised. Percutaneous transhepatic cholangiography is useful in such situation in visualizing the proximal end of the duct but it is likely to be less successful in patients whose intrahepatic ducts are not dilated in the initial stages [12]. MRC is an alternative investigation of choice when available.

### 14.3. Major Duct

Injury to a major duct (right hepatic duct/CHD or CBD) has more serious consequences. In the event of this unfortunate incidence, further management including assessment would depend on the availability of expertise [1, 2, 6].

### 14.4. Expertise Available

In an ideal situation, a trained biliary surgeon with adequate experience in reconstructive biliary surgery should carry out the repair. The procedure should be converted to an open operation, and the injury should be repaired as detailed subsequently.

### 14.5. Partial Injury

A lateral/incomplete injury (involving partial circumference of the duct) may be repaired with fine (4-0/5-0) suture of vicryl/PDS. Some recommend the placement of a T tube as a stent [23]. However, the placement of a T tube in an undilated normal size duct may be difficult and frustrating and could potentially aggravate the injury [23].

### 14.6. Complete Injury

If the duct has been divided, it is important to assess if there is associated loss of a segment of the duct as happens in the classical lap cholecystectomy injury [17]. This happens when the CBD is first clipped and divided mistaking it for the cystic duct. CHD is then encountered and divided again.

The ideal management of a complete transection of the bile duct is the restoration of the biliary enteric continuity with a Roux-en-Y hepaticojejunostomy [24]. When the bile duct has been divided without excision of a segment, a primary end to end anastomosis of the cut ends of bile duct has been described. This procedure had fallen into disrepute after a report stating that almost half of such repairs developed into strictures that later required hepaticojejunostomy [25]. A recent report from the Amsterdam Medical center, however, has revived interest in this option. Between 1990 and 2006, 56 BDIs were managed with anastomosis (49 with a T tube) [26]. These were followed with a combination of endoscopic and radiological intervention as needed. The authors reported more than 90% stricture free rates during a mean followup of 7 years [26]. A distinct advantage of this procedure is that it maintains the normal biliary drainage into the duodenum and avoids the risk of reflux associated cholangitis and stricture following hepaticojejunostomy. Another advantage of the repair is that the stricture that might result is usually of a low variety (Bismuth Type 1 or 11). These are more easily repaired surgically in the event of failure of endoscopic and radiological intervention.

### 14.7. Expertise Not Available

In most of the situation expertise for reconstruction is not available and in such situations no attempt must be made to repair the injury. Repairs done by inexperienced surgeons are likely to fail [27]. In addition, repair after a previous attempt even if done by an expert biliary surgeon is less likely to be successful [28]. When expertise to repair is not immediately available, the safest option (in the interest of both the patient and surgeon) is to irrigate the area with copious amounts of solution, observe and record the operative findings and place two large/wide bore (28 French) drain in the subhepatic fossa [29, 30]. This will drain the bile from the injured duct and prevent the formation of a bilioma. Omentum if available may also be placed in the subhepatic fossa. This can be accomplished laparoscopically and there should be no need to convert to laparotomy. This will result in a controlled external biliary fistula, thus preventing peritoneal sepsis [24–27]. Postoperatively an endoscopic papillotomy may be performed and a stent placed in the CBD in cases of partial injury to decompress the bile ducts [24, 28]. The external biliary fistula may eventually close without any biliary obstruction in case of partial injury. In some cases especially those with complete injury, the biliary fistula may not close and repair will need to be performed using the undilated proximal ducts [24–27]. More often a biliary stricture develops (with dilated proximal ducts) which will require a hepaticojejunostomy. Placement of a tube into the proximal end of the divided duct to convert the BDI into a controlled external biliary fistula is attempted by some. The attempt to place a catheter into the injured nondilated proximal duct during the course of a laparoscopic cholecystectomy may, however, cause further injury to the CHD, particularly when performed by an inexperienced surgeon. Clipping of the divided duct is sometimes performed with intent to prevent bile leak and allow the injured duct to stricture resulting in the proximal duct dilatation which facilitates a hepaticojejunostomy [31]. This is rarely successful because in the majority of cases the clipped or ligated ducts sloughs, thus causing the inevitable bile leak and resulting in the injury becoming even more proximal. Moreover, the clip (or ligature) also interferes with the blood supply and causes ischaemic injury [31].

### 14.8. Missed Injury

In the majority of cases (more than 60%), the biliary injury is unrecognized at laparoscopic cholecystectomy [20, 32]. A high index of suspicion is essential to recognize biliary injury (leak or transaction of CBD) in the early postoperative period. In a study of 207 patients with postoperative bile duct leak who underwent ERCP, the most common site of leak included cystic duct stump (78%), a peripheral right hepatic duct (Luschka 13%), and other sites like common bile duct and T tube insertion point (9%) [12]. The leak could either be low grade (LG) where the leak is noted only after the opacification of the intrahepatic biliary radicles with contrast following ERCP or a high-grade leak (HG) when the leak is observed fluoroscopically before intrahepatic duct opacification [12]. The later is considered more significant as the spillage of contrast occurs with minimal injection pressure and before the opacification of the ductal system. Patients with LG leak are effectively managed by sphincterotomy alone or placement of nasobiliary tube or stent placement, and it could achieve reduction in pressure gradient and allow closure of leak in >90% [12]. HG leak however would require stent placement with probably bridging the site of leak-like cystic duct stump leak. Decision of stent placement is however determined by the severity of leak rather than site of leak [12].

 If there is no bile leak, the patients may not have any symptoms and signs in the early postoperative period and may develop jaundice after an uneventful discharge from the hospital. Therefore, a follow-up visit approximately 1 to 2 weeks after cholecystectomy is desirable. Some BDIs especially ischaemic may present several months or even years after cholecystectomy [5, 6, 8, 17, 30]. The management of injury detected after discharge from the hospital should be performed at a center with appropriate expertise outlined previously.

The procedure of choice for repair of a major duct injury or stricture is a hepaticojejunostomy [24–28].

### 14.9. Hepaticojejunostomy

Hepaticojejunostomy is preferred to choledochoduodenostomy as the latter is prone for complications due to reflux cholangitis [5, 9, 33]. Hepaticojejunostomy with Roux-en-Y anastomosis reduces the tension of anastomosis and provides good blood supply and is the preferred option to treat duct transection injury [5, 9, 26–28, 33]. It is also the procedure of choice to treat duct defect and strictures.The outcome is significantly influenced by the surgical technique especially when the duct is not dilated [27, 28]. The outcome is better when one layer end to end anastomosis with 5-0 absorbable suture is carried out with the loop for bile drainage longer than 50 cms to avoid reflux and infection [5, 9, 26–28, 33]. The dead tissue at the end of the duct should be debrided [26, 28]. Some would place a temporary stent tube through the area of reconstruction when the duct is small. The tube helps to perform the anastomosis while permitting to perform cholangiography to check in a week or so, and it may serve as a drain if the anastomosis is temporarily leaking. The use of a transanastomotic stent is, however, debatable [25, 26]. Those who favour stenting and decompression of biliary tract claim a lower probability of postoperative stricture as it ensures a minimal size of anastomosis as healing occurs and inflammation settles down and allows easy access for diagnostic and therapeutic intervention in postoperative period [27, 29]. But others including the ones with experience from liver transplantation suggest that the use of stents is not necessary and may be harmful as it may predispose to the risk of cholangitis and prolong it once it develops [30]. If stents are placed they are maintained for 3 months [27, 29]. In patients with stricture at or above the bifurcation, the hilar plate is mobilized to obtain an adequate length of the left hepatic duct [27, 29, 31]. This would ensure an adequate width of the anastomosis and facilitate accurate mucosa to mucosa anastomosis which is essential for satisfactory reconstruction. Recurrent anastomotic stricture if it occurs as delineated on MRC is best managed by percutaneous intervention in the form of balloon dilation [29, 30, 32].

### 14.10. Bile Duct Injury Associated with Vascular Injury

This is an injury where both the bile duct and hepatic artery and/or portal vein are involved [9, 27, 34]. The bile duct injury may be caused by operative trauma, be ischaemic in origin or both, and may or may not be accompanied by various degree of hepatic ischaemia [9, 27, 34]. Right hepatic artery (RHA) vasobiliary injury (VBI) is the most common variant. Injury to RHA is likely to extend the biliary injury to a higher level than the gross observed mechanical injury. VBI results in slow hepatic infarction in about 10% of patients [9]. Occlusion of RHA without a concomitant biliary or portal vein injury rarely results in clinically significant ischaemia to liver or bile duct. Injury to RHA or its branches has been reported in 7% of the cadaver that underwent LC in life, and yet there was no abnormality of the liver or bile ducts [35]. Repair of the artery is rarely possible as it must be performed within a short time of the occurrence of the injury ideally within hours and the injury is frequently too severe to repair [9, 34]. Hence, repair of artery is rarely feasible, and the benefit is unclear. Injuries involving the portal vein or common or proper hepatic artery are much less common but have more serious effects including rapid infarction of the liver [9, 34]. Routine arteriography is recommended in patients with biliary injury if early repair is contemplated. The repair of biliary injury associated with vascular injury is often delayed [9, 34]. This is due to the consequence of poorer results of early in comparison to delayed repair. This is attributed to the fact that bile duct necrosis can progress after biliary injury and may not reach a stable state for about 3 months [9, 34]. Therefore, performing a bile duct reconstruction soon after injury in the presence of RHA injury risks repairing at a site on the bile duct that may appear viable but in fact is destined to be fibrotic. As the RHA lies at the level of the mid common hepatic duct, biliary injuries that occur in association with RHA injury are likely to be at the level of E1-E2 [9, 34]. Portal vein is much less vulnerable to injury during LC than RHA; consequently, there are only 16 such cases reported of portal vein injury associated with major biliary injury with death resulting in 50% of patients with infarcted liver [9].

## 15. Results of Repair

The goal of surgical repair of the injured biliary tract is the restoration of a durable conduit and the prevention of short- and long-term complications, such as biliary fistula, intraabdominal abscess, biliary stricture recurrent cholangitis, and secondary biliary cirrhosis [5, 6, 8, 10]. The diagnostic evaluation of the patient with biliary injuries should include accurate determination of the biliary anatomy. Suspected intra-abdominal abscess formation or vascular injury can be detected by computed tomography or magnetic resonance cholangiography. The preoperative determination of biliary anatomy is of outmost importance for better outcome as poor outcome following repair was reported in 96% of patients without cholangiography and in 69% in patients with incomplete cholangiographic data [13]. The overall long-term success rate when performed in specialized center is >90%, and the mortality rate was 0 to 3.2% [26, 28, 31, 33, 36]. The factor that influence the long-term outcome after hepaticojejunostomy include the presence of active peritonitis at the time of repair, the combination of bile duct and vascular injury, the level of injury at or above the biliary bifurcation, and the number of previous operations [5, 9, 26, 28, 36]. Also of outmost importance is to whether the surgery was performed by the primary surgeon or performed at specialized center as the reported success rate in them is 35% and >90%, respectively [31]. The factors influencing mortality include the number of previous operations, a history of major infection, the site of stricture, preoperative serum albumin concentration, and the presence of liver disease and portal hypertension [25, 26]. The Johns Hopkins group had reported their results of repair of 142 BDIs performed between 1990 and 1999 with a mortality rate of 0.6%. At a mean followup of 55 months, excellent/good results were obtained in 91% of the patients. Thirteen patients had anastomotic failure and 10 of these were salvaged by reintervention [31]. In another study of 300 strictures performed between 1989 and 2006 the mortality rate was 1.3%. Among the 225 followed up for more than 2 years, 91% had excellent/good outcome whereas 11 patients required re-intervention for failure [37]. Two thirds of recurrence occurs within 2 years but stricture recurrence after 10 years have also been reported [38].

## 16. Legal Implications

Injury to the bile duct at laparoscopic cholecystectomy is not always a result of negligence. It is a known complication of the procedure. However, the patient should have been informed about this during the consent process and adequate measures should have been taken in early detection and appropriate management. A review of the data provided by NHS litigation authority (NHSLA) to assess the prevalence and outcome claims reported that the claim due to CBD injury following LC was the maximum constituting 41% of the total followed by bile leak (12%), bowel injury (9%), haemorrhage (9%), and fatality (9%) [7]. The highest proportion of successful claims are for bile duct injury ranging from 86% (UK report) [7] to 18% (Dutch report) [39] and 8% (USA report) [40]. Successful claim or settlement was associated with death of the patient, patient loss of income and untimely delay in the detection and inappropriate referral and management as these were considered as negligence [7, 38, 39]. When relaparotomy was performed in the initial centre the financial compensation was doubled [7]. Proper training of the surgeon in laparoscopic cholecystectomy, detailed informed written consent before operation, documentation of the procedure (preferably video recording but at least detailed operative notes) truthful communication of the mishap to the patient family, and timely referral of the patient and family to an appropriate facility will defend the surgeon in most of the cases [7, 38, 39]. It is well documented that maximum compensation is awarded to the patient in case of delay in detecting the complication and if an unsuccessful repair was attempted by an inexperienced primary surgeon [7, 38, 39].

## 17. Conclusion

LC which is a gold standard therapeutic option for symptomatic cholecystolithiasis is however associated with increased risk of CBD injury compared to open approach. While local factors including acute cholecystitis, fibrosed contracted gall bladder, anatomic anomalies are some of the contributing factors, significant number of cases are associated with the so called “easy” cholecystectomy performed by an inexperienced surgeon. Adequate training in laparoscopic surgery, proper delineation of biliary anatomy in Calot's triangle, judicious use of electrocautery, avoiding blind application of clips and cautery, performing intra-operative cholangiogram, and converting to open procedure in the event of failure to progress or uncertain anatomy would go a long way in significantly reducing this mishap. While simple leaks from cystic duct stump or minor duct are effectively treated by ERCP sphincterotomy, major duct injury is best dealt with hepaticojejunostomy particularly performed in specialized center. Successful litigation claims are associated with delay in detection of this complication, attempt to repair at the primary center without specialized biliary surgery service, and failure to refer to an appropriate center. Failure of appropriate management will increase health care expenses, lead to impaired quality of life, and in unfortunate cases may even lead to death.
